# A Modeling Method for Thermal Error Prediction of CNC Machine Equipment Based on Sparrow Search Algorithm and Long Short-Term Memory Neural Network

**DOI:** 10.3390/s23073600

**Published:** 2023-03-30

**Authors:** Ying Gao, Xiaojun Xia, Yinrui Guo

**Affiliations:** 1 School of Computer Science and Technology, University of Chinese Academy of Sciences, Beijing 100049, China; 2 Shenyang Institute of Computing Technology, Chinese Academy of Sciences, Shenyang 110168, China; 3 School of Mathematics and Computer Sciences, Chifeng University, Chifeng 024000, China

**Keywords:** thermal error prediction, temperature-sensitive points screening, FCMCA, SSA-LSTMNN, CNCME

## Abstract

To better solve the problem of thermal error of computerized numerical control machining equipment (CNCME), a thermal error prediction model based on the sparrow search algorithm and long short-term memory neural network (SSA-LSTMNN) is proposed. Firstly, the Fuzzy C-means clustering algorithm (FCMCA) is used to screen the key temperature-sensitive points of the CNCME. Secondly, by taking the temperature rise data of key temperature-sensitive points as input and the corresponding time thermal error data as output, we established the SSA-LSTMNN thermal error prediction model. The SSA is used to optimize the parameters of LSTMNN and make its performance play the best. Taking the VMC1060 vertical machining center as the research object, we carried out the experiment. Finally, the prediction effect of the proposed model is compared with the article swarm optimized algorithm and LSTM neural network (PSOA-LSTMNN), the LSTMNN, and the traditional recurrent neural network (TRNN) model. The results show that the average values of the predicted residual fluctuations of the SSA-LSTMNN model are all more than 44% lower than those of the other three models under different operating conditions, which has a strong practicality.

## 1. Introduction

Computerized numerical control machining equipment (CNCME) is known as the “Industrial mother-machine tool” of the equipment manufacturing industry. As a heavy-duty, high-precision, general-purpose machine tool, CNCME is the core production equipment for many high-tech industries that support economic development. It can be said that the research and development capabilities of high-end CNCMEs have already become a yardstick to measure the level of the national manufacturing industry. China’s “14th Five-Year Plan” also points out that, from 2021 to 2025, the overall goal of the development of the CNCME industry is to achieve the industrial basis of high-grade and industrial chain modernization in upmarket CNCME products by 2025, and the industrial layout is to be balanced and reasonable; the domestic CNCME, cutting tools, CNC-systems and functional components are to have the basic support and meet the needs of domestic economic development; key CNCME products are to be close to or up to the international advanced level; and the proportion of domestic high-end products in the market will have steadily increased, cultivating many independent property rights and international competitiveness of brand-name enterprises and products. Therefore, the importance of CNCME in the manufacturing field is self-evident. However, machining accuracy is the heart of CNCME. Achieving high workpiece accuracy is the long-term goal of machine tool designers [1]. If one wants CNCME to work efficiently, one must ensure that the heart reaches the best state. The main factors affecting machining accuracy are machining errors, including geometric errors, thermal errors, and errors caused by cutting forces [2]. It has been shown that under the conditions of high-speed and high-precision machining, the error caused by the thermal deformation of machine tools accounts for the largest proportion of the overall error of CNCME and that thermal deformation has been the most important factor affecting the machining accuracy of CNCME [3]. In precision machining, thermal error accounts for more than 70% of the total error [4]. Thermal error compensation is an effective method to eliminate the thermal error of machine tools [5]. Therefore, if the predicted value of thermal error can be accurately obtained, then the error analysis and compensation based on the predicted value is of substantial significance to eliminate the thermal error of CNCME.

**Figure 1:**
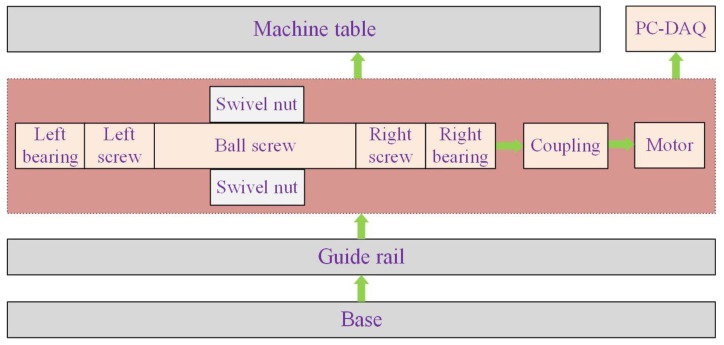
Diagram of internal heat source components of CNCME.

The thermal error of CNCME means that the machine tool is affected by internal and external heat sources during processing, and the temperature changes in various parts of the machine tool cause thermal expansion of various parts. Various thermal displacements such as stretching, bending, and twisting caused by thermal expansion change the relative position between cutting tools and workpieces, thereby reducing the machining accuracy of the machine tool and causing errors [6]. The main reason for this is that the balance between the internal heat source and the external heat source of the machine tool is broken, and the resulting temperature gradient causes thermal stress inside the machine tool, resulting in a certain degree of thermal deformation of the machine tool components. The internal heat source refers to the heat loss caused by friction of the main parts of the machine tool, such as bearings, nuts, guides, motors, etc., during the working process of the machine tool and the heat generated during the tangent movement of the machine tool [7]. The structure diagram of the main components of the heat source inside the machine tool is shown in Figure 1. The external heat source mainly refers to the heat impact on the machine tool caused by the change of ambient temperature and the change of the day and night temperature of the machine tool workshop. The influence of the internal heat source on the thermal error of the machine tool is much greater than that of the external heat source. The processing conditions of CNCME are complex, and many conditions that occur during processing are unpredictable, leading to the characteristics of different temperature rise intensities and uneven distribution of the internal heat source, which make the thermal error of the machine tools very complex, showing a non-linear feature [8]. The methods of reducing the thermal error of machine tools include the hardware method and software method. The hardware method is the error prevention method, and the software method is the error compensation method [9]. The hardware method prevents the excessive thermal error of the machine tool by improving the manufacturing accuracy and installation accuracy of the machine tool, designing the machine tool structure symmetrically, separating heat sources, strictly controlling the machine tool structure, and other means. However, this method is high in cost and low in efficiency, which is difficult to implement due to the limitation of the hardware structure [10]. In the software compensation method, state-of-the-art technology is used to reduce CNC machine tool thermal errors, and it belongs to key intelligent functions of modern machine tools [11]. The software method is to convert the thermal error compensation value into a control command that can be recognized by the CNC system and control the CNCME to perform additional motions in the opposite direction of the thermal error to eliminate or reduce the thermal error [12]. The software method is economical and efficient to implement and has become the mainstream method to improve the machining accuracy of CNCME [13]. The software method requires obtaining an accurate thermal error compensation value and then performing thermal error software compensation for this compensation value. Therefore, establishing an accurate thermal error prediction model and obtaining an accurate thermal error prediction value is the key to effective thermal error software compensation [14]. Before establishing the thermal error prediction model, the temperature data of each temperature measuring point should first be obtained. Affected by internal and external heat sources, the temperature rise data of each temperature point of the machine tool is collinear and time varying, and the temperature field distribution is very complex [15]. To grasp the temperature change of the machine tool, it is a common practice to arrange a large number of temperature sensors in all parts of the machine tool that may generate heat to collect real-time temperature change data of the machine tool. However, with the increase in the number of sensors, not only will the cost and workload of the experiment increase significantly, but also, too many temperature measuring points and wiring will cause problems such as insufficient data interface, measurement point coupling, poor model robustness, etc. [16]. Therefore, it is the basis for the implementation of thermal error compensation to select the thermal key point that best reflects the temperature state of the machine tool from a large number of temperature measurement points [17]. Understanding the heat source distribution of the machine tool, finding out the key heating parts, and collecting the data of the key thermal temperature points are of significance for the prediction and modeling of the thermal error of CNCME. After obtaining the temperature data of the representative hot spots, the neural network model, which is a popular tool with a strong nonlinear system simulation function, can be used to carry out self-learning of the data so that the thermal error of CNCME can be well predicted.

Zhang et al. [18] used the fuzzy clustering method to divide the 29 temperature measurement points of the CNCME into 5 groups and introduced the Slice Inverse Regression (SIR) model for thermal error modeling, successfully reducing the axial thermal error from 43 μm to 7 μm. Li et al. [19] introduced the K-harmonic means (KHM) clustering algorithm in the thermal error field to select the temperature-sensitive points of the machine tool and compared it with other clustering methods to verify the stability of KHM. Than, V.T. et al. [20] presented a thermal error model for a lathe CNC machine using temperature on moving parts, and, combined with the use of multiple linear regression, the relationship between temperature and thermal error is modeled. Krstic, V. et al. [21] presented a prediction of the total friction torque and temperature by axial angular contact ball bearings designed for threaded spindles using ANN (artificial neural network). Abdulshahed, A.M. et al. [22] combined the cuckoo search algorithm and gray correlation model to design a thermal error prediction model for CNC machine tools; the results show that there is good agreement between predicted and experimental thermal errors. Fu et al. [23] proposed a chicken swarm optimization algorithm-based radial basic function (CSO-RBF) neural network to deal with the nonlinear relationship between temperature variables and thermal errors and adopted a K-means clustering and radial basis function (KC-RBF) neural network based on a correlation analysis method to screen the best combination of temperature-sensitive points, and, finally, they established a thermal error model with a strong prediction effect. Yang et al. [24] used K-means clustering and gray model (GM) to select temperature-sensitive points; in addition, they proposed an improved grey wolf optimizer (IGWO) and an adaptive neuro-fuzzy inference system (ANFIS) accurate thermal error model. Li et al. [25] used the self-organizing map (SOM) neural network to realize the clustering of temperature measurement points and established a spindle thermal error prediction model with good prediction accuracy based on the improved particle swarm optimization and back-propagation (IPSO-BP) neural network. Li et al. [26] established a thermal error prediction model using an improved particle swarm optimization (PSO) algorithm and reduced the number of machine tool temperature measuring points from 10 to 4 using K-means clustering; the results show that the model has good stability. Jia et al. [27] proposed a temperature-integrated regression method to eliminate the collinearity between temperature data. Cao et al. [28] proposed a thermal error prediction model based on linear correlation research of multiple linear regression and principal component analysis, and the experimental results show that the axial prediction accuracy of this method can reach 1.099 μm. Yue et al. [29] proposed a thermal error prediction model based on an adaptive chaotic particle swarm optimization algorithm and reduced the number of temperature measuring points from 12 to 6 by using fuzzy clustering and a gray correlation algorithm, with the final modeling accuracy reaching more than 90%. Due to the significant impact of Industry 4.0 on the machine tool industry and the high expectations of China’s “14th Five-Year Plan” for the machine tool manufacturing industry, the thermal error modeling of CNCME has encountered unprecedented challenges. Existing thermal error modeling methods are far from adequate in handling large-scale time-series problems. The existing thermal error prediction models were often established by analyzing the mapping relationship between the temperature of each part of the machine tool at some specific time and the thermal error at the corresponding time while ignoring the correlation between the thermal error and the temperature rise data at the historical time. The thermal hysteresis effect and thermal deformation accumulation make the thermal error not only depend on the temperature characteristics of the current time but also have an inseparable relationship with the temperature characteristics of the past continuous time [30]. Therefore, in order to establish a more accurate thermal error prediction model, a long short-term memory neural network (LSTMNN) with memory cells is introduced in this paper. The temperature rise data of CNCME are a continuous physical quantity that changes with time. The temperature rise data at the next time are closely connected with the temperature rise data at the past historical time. The temperature data at a certain point is dependent on the previous temperature data, the long-term and short-term memory network based on time series introduces more long-term memory cells and short-term memory cells about the last time data than the ordinary traditional neural network, which makes the LSTMNN have a better effect on data processing with continuous characteristics in time and makes it more suitable for establishing the prediction model of thermal error of CNCME. This paper adopts the thermal error prediction method combining FCMCA and LSTMNN. First, the FCMCA was used to screen the temperature measuring points of CNCME, and the key thermal temperature-sensitive points were selected. Then, the temperature data of the selected key thermal temperature-sensitive points were used as the input of the LSTMNN model, and the thermal error data collected at the corresponding time were used as the model output to train the thermal error prediction model. The temperature data and thermal error data of different working conditions were collected on the VMC1060 vertical machining center for experiments. Finally, when compared with the traditional recurrent neural network (TRNN) thermal error prediction model, the superiority of the method proposed in this paper was further verified.

## 2. Thermal Error Modeling Principle

### 2.1. Key Temperature-Sensitive Points Screening

To eliminate the temperature redundant data that would affect the thermal error modeling, solve the collinearity problem of temperature measurement point data, improve the prediction performance of the model, reduce the amount of calculation, and obtain more representative characteristics of machine tool temperature point data that can represent the changes of machine tool thermal error, it was necessary to select the thermal critical temperature-sensitive points of the machine tool.

FCMCA is a clustering method based on partition. It combines the fuzzy mathematics theory with the K-means clustering algorithm, making it an improvement of the K-means clustering algorithm. General clustering methods, such as K-means clustering, simply divide data into unrelated classes but do not consider the correlation between data. For the complex temperature field of the CNCME bed, the temperature point data between components may have a strong correlation. For example, the temperature data of each temperature point on a bearing or of each temperature point of multiple bearings are very similar, and there is no obvious boundary. In general clustering methods, all points whose differences are not obvious are rigidly counted in one category, while FCMCA strictly calculates the membership degree of each point from each cluster center, generates a membership degree matrix, the membership degree value of a certain point to which membership center is the largest, and which cluster center this point belongs to. Strictly speaking, FCMCA has higher accuracy, fewer clustered error samples, and a shorter running time. Therefore, this paper adopts the FCMCA to cluster and divide the temperature points of CNCME.

FCMCA is a typical fuzzy clustering algorithm, and the membership degree is used to represent the possibility that each element to be classified belongs to a certain cluster center. For example, if n, data, are divided into C, categories, the objective function of FCMCA is:

(1) $$J\left(u_{ij},C_{i}\right)=\sum_{i=1}^{K}\sum_{j=1}^{N}u_{ij}^{m}\|x_{j}-C_{i}{\|}^{2}\;$$

among them, *N* represents the total number of samples to be clustered, and *K* represents the number of centers of clusters, which means that *N* samples are to be clustered into *K* categories. $C_{i}$ represents the center of the ith cluster; $\left\|x_{j}-C_{i}\right\|$ represents the Euclidean distance from the *j*-th sample point to the *i*-th cluster center, $C_{i}$; *m* is the fuzzy weight; and the value range is generally between (1.5, 2.5), for which, here, *m* = 2 is sufficient. $u_{ij}$ represents the degree of membership (or probability) from the *j*-th point to the *i*-th center, and $u_{ij}$ needs to meet a constraint condition as Equation (2); that is, the sum of probabilities from point *j* to all cluster centers from 1 to *K* must be 1.

(2) $$\sum_{i=1}^{K}u_{ij}=1\;\;\;\;\;\;j=1,2,\ldots ,N$$

To find the minimum value of the objective function J, Lagrangian factors need to be introduced to construct a new objective function as follows:

(3) $$\bar{J}(u_{ij},C_{i},\lambda_{j})=\sum_{i=1}^{K}\sum_{j=1}^{N}u_{ij}^{m}\|x_{j}-C_{i}{\|}^{2}-\sum_{j=1}^{N}\lambda_{j}\left(\sum_{i=1}^{K}u_{ij}-1\right)$$

among them, $\lambda_{j}\left(j=1,2,\ldots ,N\right)$ is the Lagrangian factor. To find the minimum value of the objective function, $\bar{J}$, first, let the above Equation (3) take the partial derivative of $u_{ij}$, and then set the derivative value to 0:

(4) $$\frac{\partial \bar{J}}{\partial u_{ij}}=0$$

Equation (5) is obtained by (4):

(5) $$u_{ij}=\frac{1}{\sum_{q=1}^{K}{\left(\frac{\left\|x_{j}-C_{i}\right\|}{\left\|x_{j}-C_{q}\right\|}\right)}^{\frac{2}{m-1}}}$$

In (5), “*q*” and “*i*” express the same meaning, but to distinguish from $u_{ij}$ on the left side of the equal sign, the “*i*” on the right side of the equal sign is replaced by “*q*”.

Next, let Equation (3) take the partial derivative of $C_{i}$ and then take the extreme value as Equation (6):

(6) $$\frac{\partial \bar{J}}{\partial C_{i}}=0$$

$C_{i}$ is obtained as follows:

(7) $$C_{i}=\frac{\sum_{j=1}^{N}u_{ij}^{m}x_{j}}{\sum_{j=1}^{N}u_{ij}^{m}}$$

From (5) and (7), after obtaining $u_{ij}$ and $C_{i}$ and substituting them into Equation (3), the value of the objective function $\bar{J}$ is obtained, as shown in Equation (8). According to the above Equations (1)–(8), the process is as follows: iterate continuously until the objective function values of the two-time *t* and *t* − 1 satisfy (9), then stop the iteration (ε is the set threshold) and obtain the optimal function value ${\bar{J}}^{t}$ at time *t*. Alternatively, set the number of cluster centers *C* ((*C* = 1, 2, … *N*), where *N* is the number of cluster samples), and find the derivative value of the objective function of each clustering, $\left|{\bar{J}}^{t^{\prime }}\right|$; when *C* takes the minimum value and satisfies Equation (10), the optimal function value, ${\bar{J}}^{t}$, and the optimal number of cluster centers are obtained.

(8) $${\bar{J}}^{t}=\sum_{i=1}^{K}\sum_{j=1}^{N}u_{ij}^{m}\|x_{j}-C_{i}{\|}^{2}$$

(9) $$\left|{\bar{J}}^{t}-{\bar{J}}^{t-1}\right|\leq \varepsilon$$

(10) $$\left|{\bar{J}}^{t^{\prime }}\right|\leq \varepsilon$$

The steps of the FCMCA to screen the thermally critical temperature-sensitive points of CNCME can be summarized into the following five steps:

1. Initialize the membership values, $u_{ij}$.
2. Calculate the cluster centers by Equation (7).
3. Update $u_{ij}$ by Equation (5).
4. Compute the value of the objective function ${\bar{J}}^{t}$ by Equation (8).
5. If $\left|{\bar{J}}^{t}-{\bar{J}}^{t-1}\right|\leq \varepsilon$ or $\left|{\bar{J}}^{t^{\prime }}\right|\leq \varepsilon$, then stop; otherwise, return to step 2.

### 2.2. Long Short-Term Memory Neural Network

In this paper, the LSTMNN is introduced in the establishment of the thermal error prediction model because the traditional neural network has the problem of gradient explosion and gradient disappearance; the gradient disappearance problem is especially serious, and at this time, the LSTMNN came into being. The TRNN can realize short-term memory through a memory cell to predict continuous data, but when the sequence of continuous data is too long, that is, when the amount of data is too large, it causes the memory of the TRNN to expand along the time axis for too long. During back-propagation, the data are too long, the time step is too long, the period becomes larger, and the multiplied power becomes larger. If the parameter of back-propagation is too small, for example, the parameter *W* is less than 1, which is 0.9, as the multiplication power increases during the back-propagation process, the multiplication result tends to zero, which causes the problem of gradient disappearance. If the parameter is too large during back-propagation—for example, *W* is a number greater than 1, and the value of *W* is 1.1—with the continuous multiplication in the process of back-propagation, the final product continues to increase and tends to infinity, which leads to the gradient explosion problem. The LSTMNN was generated to solve the problem of gradient disappearance and gradient explosion of TRNN.

Compared with the TRNN, the LSTMNN has three more controllers: input control, forgetting control, and output control. The TRNN tries to remember all the information, whether it is useful information or useless information. However, the LSTMNN is designed with a memory cell, which has the function of selective memory. It can choose to memorize important information, filter out redundant and useless noise information, and reduce the burden of memory. Three thresholds and two states are introduced into the hidden layer structural unit of the LSTMNN, namely the input threshold, $i_{t}$; the forgetting threshold, $f_{t}$; the output threshold, $o_{t}$; the cell state, $C_{t}$, representing long-term memory; and the candidate state, $\tilde{C_{t}}$, waiting to be stored in long-term memory (indicates new knowledge or new memory that has been summarized). $h_{t}$ is the memory for short-term memory. These values are calculated as follows:

(11) $$i_{t}=\sigma \left(W_{xi}\cdot x_{t}+W_{hi}\cdot h_{t-1}+b_{i}\right)$$

(12) $$f_{t}=\sigma \left(W_{xf}\cdot x_{t}+W_{hf}\cdot h_{t-1}+b_{f}\right)$$

(13) $$o_{t}=\sigma \left(W_{xo}\cdot x_{t}+W_{ho}\cdot h_{t-1}+b_{o}\right)$$

(14) $$C_{t}=f_{t}*C_{t-1}+i_{t}*\tilde{C_{t}}$$

(15) $$\tilde{C_{t}}=\tan h\left(W_{xc}\cdot x_{t}+W_{hc}\cdot h_{t-1}+b_{c}\right)$$

(16) $$h_{t}=o_{t}*\tanh\left(C_{t}\right)$$

**Figure 2:**
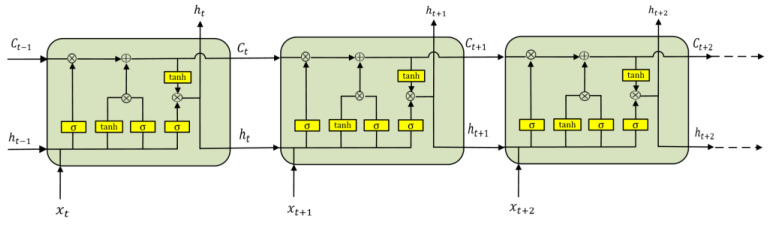
LSTMNN structural unit diagram.

$x_{t}$ is the input feature of the current moment; the memory, $h_{t}$, represents the short-term memory; $h_{t-1}$ is the short-term memory of the previous moment; $W_{xi},\;W_{xf},\;\mathrm{and}$ $W_{xo}$ are the parameter matrices to be trained for $x_{t}$; $W_{hi}$, $W_{hf}$, and $W_{ho}$ are the parameter matrices to be trained for $h_{t-1}$; and $b_{i}$, $b_{f}$, and $b_{o}$ are the bias items to be trained. The three thresholds, $i_{t}$, $f_{t}$, and $o_{t}$, are all functions about $x_{t}$ and $h_{t-1}$, as shown in Equation (11) to (13). σ represents the sigmoid activation function, making the threshold range between 0 and 1. Cell state, $C_{t}$, represents long-term memory. The cell state is the result of multiplying the long-term memory of the previous moment by the forgetting threshold, plus the result of multiplying the new knowledge summarized at the current moment by the input threshold, as shown in Equation (14). The candidate state, $\tilde{C_{t}}$, represents the newly summarized new knowledge to be stored in the cell state and is also a function of the input feature at the current moment and the short-term memory at the previous moment, as shown in Equation (15). Short-term memory, $h_{t}$, is a part of long-term memory, $C_{t}$, so it is a function of long-term memory, which is the result of the cell state being activated by the activation function tanh and then filtered by the output threshold, as shown in Equation (16). When there is a multi-layer recurrent network, the input to the second-layer network is the essence obtained by removing the useless information from the first field network. The structural unit diagram of the LSTMNN is shown in Figure 2.

### 2.3. Principle of Sparrow Search Algorithm

Since the parameters of the neural network are optimized by some algorithms, the network performance becomes more stable, the convergence speed is faster, and the accuracy is higher. Therefore, more and more experts and scholars are paying attention to solving the parameter optimization problem of neural networks through optimization algorithms. In 2020, a new intelligent optimization algorithm based on population, the sparrow search algorithm (SSA), was proposed [31]. The SSA was inspired by the predation phenomenon of sparrows in nature. Sparrows are very clever and have a strong memory and group consciousness. They can have a clear division of labor in the process of finding food, and some sparrows can signal their partners to go to a safe place when there is danger. Sparrows generally divide population members into discoverers, joiners, and scouts in the process of hunting. The discoverer seems to play the role of the leader of social animals. The discoverer has a strong search ability. The discoverer first finds the area where the food is and directs other members to the area to forage together. The joiners join the group through the guidance of the discoverer and look for food nearby. The scout is the member who is the most alert to the surroundings among all sparrows and has strong vigilance. If there is danger around the foraging area, such as possible natural enemies, the scout will send out a warning signal to inform other members to evacuate, and the population will make corresponding anti-predation behavior when they are aware of the danger. The implementation of the sparrow search algorithm is as follows:

1. Initialize the population.

Assuming that there are *N* sparrows in an *M*-dimensional search space, the group composed of *N* sparrows can be expressed as follows:

(17) $$X={\left[\;\;\begin{array}{c} X_{1}\\ \vdots \\ X_{i}\\ \vdots \\ X_{N} \end{array}\;\;\right]}_{N\times M}={\left[\;\;\begin{array}{ccccc} x_{1,1} & \cdots & x_{1,j} & \cdots & x_{1,M}\\ \vdots & \ddots & \vdots & \ddots & \vdots \\ x_{i,1} & \cdots & x_{i,j} & \cdots & x_{i,M}\\ \vdots & \ddots & \vdots & \ddots & \vdots \\ x_{N,1} & \cdots & x_{N,j,} & \cdots & x_{N,M} \end{array}\;\;\right]}_{N\times M}\;,\;i=1,2,\ldots ,N;j=1,2,\ldots ,M;$$

where $x_{i,j}$ represents the specific position of the *i*-th sparrow in the *j*-dimensional space.

2. Determine the fitness function.

The search ability of each sparrow can be characterized by a fitness function value *f*. The fitness function values of all sparrows can be expressed as follows:

(18) $$F_{X}={\left[\;\;\begin{array}{c} F_{1}\\ \vdots \\ F_{i}\\ \vdots \\ F_{N} \end{array}\;\;\right]}_{N\times 1}={\left[\;\;\begin{array}{c} f\left(\left[x_{1,1}\ldots x_{1,j}\ldots x_{1,M}\right]\right)\\ \vdots \\ f\left(\left[x_{i,1}\ldots x_{i,j}\ldots x_{i,M}\right]\right)\\ \vdots \\ f\left(\left[x_{N,1}\ldots x_{N,j}\ldots x_{N,M}\right]\right) \end{array}\;\;\right]}_{N\times 1}\;\;\;,\;i=1,2,\ldots N;j=1,2,\ldots ,M;$$

3. Update the location of the discoverer.

Discoverers generally account for 10% to 20% of the population, with the highest fitness value and the widest search range. The location update of the discoverer is described as follows:

(19) $$X_{i,j}^{t+1}=\left\{\begin{array}{ll} X_{i,j}^{t}\cdot \exp\left(-\frac{i}{iter_{max}\cdot \varepsilon }\right), & W<S\\ X_{i,j}^{t}+P\cdot Q, & W\geq S \end{array}\right.$$

t and $iter_{max}$ represent the current and maximum iteration times of the algorithm. $X_{i,j}^{t}$ represents the position of the *i*-th sparrow in the *j*-th dimensional space in the current t-th iteration. $X_{i,j}^{t+1}$ represents the position of the *i*-th sparrow in the *j*-th dimensional space in the next *t* + 1st iteration. *W* stands for danger signal value, which generally ranges from 0 to 1. *S* represents the safety threshold, ranging from 0.5 to 1. When *W* < *S*, the feeding area is relatively safe, and sparrows can hunt in this area. When *W* ≥ *S*, it indicates that there are natural enemies or other risk factors in the current area and that sparrows need to fly out of here quickly to find food in a safer place. $\varepsilon \in \left(0,1\right.]$ is a random number. P is a random number that follows a standard normal distribution. Q is a 1 × *M* matrix with all elements of the matrix being 1.

4. Update the location of the joiner.

The updated description of the joiner’s location is as follows.

(20) $$X_{i,j}^{t+1}=\left\{\begin{array}{ll} P\cdot exp\left(\frac{X_{worstj}^{t}-X_{i,j}^{t}}{i^{2}}\right), & i>\frac{N}{2}\\ X_{bestj}^{t+1}+\left|X_{i,j}^{t}-X_{bestj}^{t+1}\right|\cdot A^{+}\cdot Q, & i\leq \frac{N}{2} \end{array}\right.$$

$X_{worstj}^{t}$ and $X_{bestj}^{t+1}$, respectively, represent the worst position and the best position of the *i*-th sparrow in the *j*-th dimensional space in the *t*-th iteration and the *t* + 1st iteration. A is a 1×M matrix where each element is randomly assigned to 1 or −1 and satisfies $A^{+}=A^{T}{\left(AA^{T}\right)}^{-1}$. When i>N/2, it means that the *i*-th joined sparrow did not follow the finder to find food and its adaptability is low; it will fly to other places to find food. When i≤N/2, it indicates that the *i*-th joining sparrow is highly adapted and will follow the discoverer’s footsteps to forage nearby.

5. Update the location of the scout.

Approximately 10% to 20% of the sparrow population are scouts, and the process of updating the location of scouts is shown below:

(21) $$X_{i,j}^{t+1}=\left\{\begin{array}{ll} X_{bestj}^{t}+\gamma \cdot \left|X_{i,j}^{t}-X_{bestj}^{t}\right|, & f_{i}>f_{g}\\ X_{i,j}^{t}+R\cdot \left(\frac{X_{i,j}^{t}-X_{worstj}^{t}}{\left(f_{i}-f_{w}\right)+\delta }\right), & f_{i}=f_{g} \end{array}\right.$$

$X_{bestj}^{t}$ is the optimal position of the *i*-th sparrow in the *j*-th dimensional space in the current iteration number, *t.* $f_{i}$ is the adaptation value of the *i*-th sparrow, and $f_{w}$ and $f_{g}$ are the current global worst and best fitness values. $R\in \left[-1,1\right]$ is a random number that controls the direction and step size of the sparrow’s movement. γ is a random number obeying normal distribution with a mean of 0 and variance of 1. δ is a very small number to avoid a denominator of 0. When $f_{i}>f_{g}$, it means that the current member is at the edge of the population and needs to change its position to avoid any danger. When $f_{i}=f_{g}$, it means that the centrally located sparrow is aware of the danger and needs to move closer to the other members to ensure its safety.

### 2.4. Thermal Error Prediction Model Based on SSA-LSTMNN

**Figure 3:**
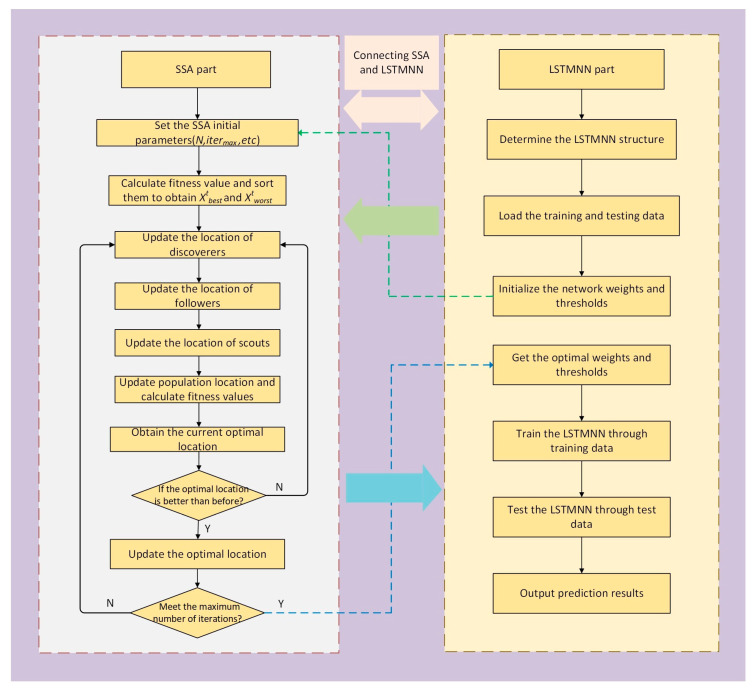
SSA-LSTMNN modeling process.

The process of optimizing the LSTMNN parameters using the sparrow search algorithm to build an SSA-LSTMNN thermal error prediction model with higher accuracy is as follows:

1. Determine the structure of LSTMNN, randomly initialize the parameters and thresholds of the neural network, and prepare the data set, including the training set and test set.
2. Set the parameters of the SSA, including:
  (1) Iteration times of the algorithm. This parameter determines the running time of the program and the stability of the model. Here, the number of iterations is set to 50.
  (2) The total number of populations, *N*, here set to 30.
  (3) The percentage of discoverers, here set to 20%.
  (4) The scout ratio, here set at 15%.
  (5) The safety threshold, S, here set to 0.8.
  (6) The dimensionality of the problem space to be optimized. Here, we wanted to optimize the number of iterations, learning rate, and the number of nodes of hidden layer units of the LSTMNN, so the algorithm dimension is set to 3.
3. Determine the fitness function. In this paper, the root mean square error (RMSE) between the predicted value of the SSA-LSTMNN model and the actual value is used as the fitness function. Calculate the fitness function value of each sparrow, and sort according to the fitness value to select the current optimal position and the worst position.
4. Update the positions of all members, including discoverers, joiners, and scouts, according to the Equations (19)–(21).
5. Obtain the current optimal value and compare it with the previous optimal value. If the current optimal value is better than the previous one, update the global optimal value; otherwise, do not update, and return to the step 4, continuing to iterate until the maximum number of iterations is met.
6. The optimal solution selected after the termination iteration of the algorithm is used to determine the parameters and thresholds of the LSTMNN for network model training. The SSA-LSTMNN modeling process is shown in Figure 3.

### 2.5. Thermal Error Prediction Model Based on PSOA-LSTMNN

**Figure 4:**
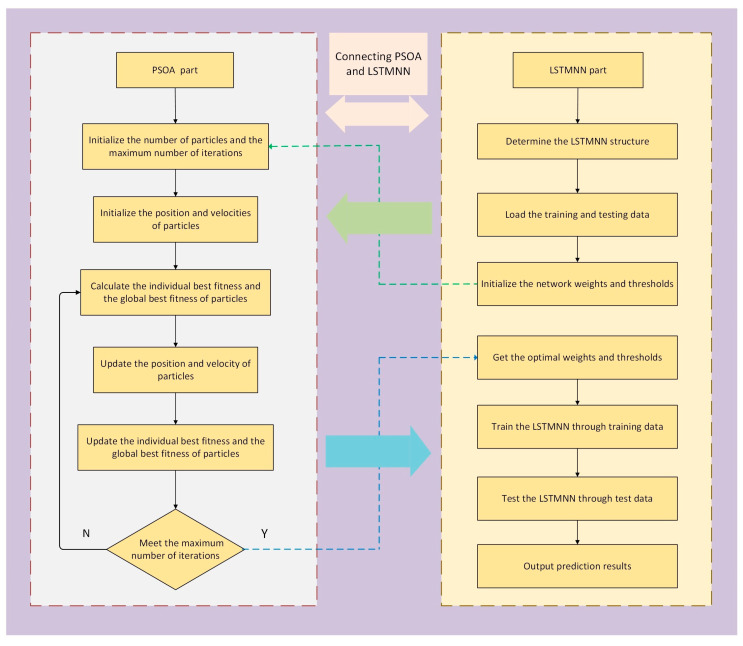
PSOA-LSTMNN modeling process.

In order to demonstrate the effectiveness and advantages of the sparrow search algorithm to optimize the parameters of the LSTMNN, we used a classical optimization algorithm, the particle swarm optimization algorithm (PSOA) [32], to optimize the parameters of the LSTMNN as well, and finally, we compared the optimization performance of the two. The modeling process of the LSTMNN with the particle swarm optimization algorithm (PSOA-LSTMNN) is as follows:

1. Determine the structure of the LSTMNN; prepare the data set, including the test and training set; and initialize the thresholds and parameters of the neural network randomly.
2. Initialize the parameters of the PSOA. Set the total number of particle swarm to 30 and the maximum number of iterations to 50.
3. Random initialization of the position and velocity vectors of each particle.
4. Determine the fitness function. The Root Mean Square Error (RMSE) of the predicted and actual values of the PSOA-LSTMNN model is used as the fitness function to calculate the individual best fitness function and the global best fitness function of the particles.
5. Update the particle’s velocity and position vectors.
6. Update the individual best-fit function values and global best-fit function values of the particles.
7. Judge whether the algorithm meets the end condition. If it does not, return to step 5 to continue iteration; if yes, continue to step 8.
8. Feed the optimal parameters optimized by the PSOA to the LSTMNN for model training. The process of thermal error prediction by the PSOA-LSTMNN model is shown in Figure 4.

## 3. Experimental Process

**Figure 5:**
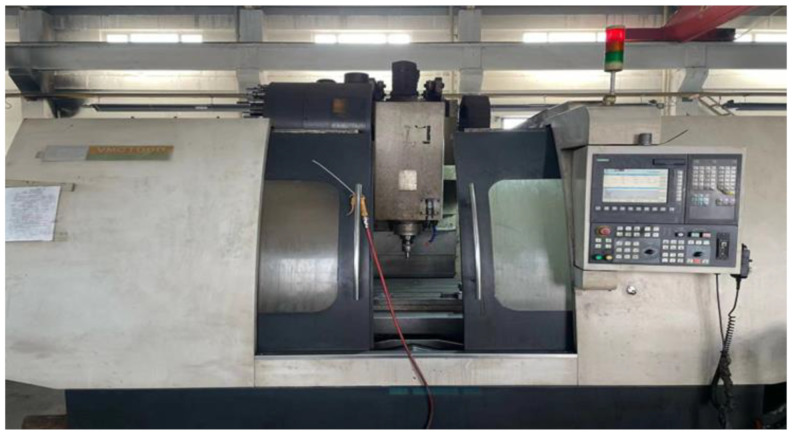
Experimental subject: VMC1060 vertical machining center.

To verify the effectiveness of the method proposed in this paper, the experimental verification was carried out on a VMC1060 vertical machining center provided by the laboratory, as shown in Figure 5. During the experiment, it was necessary to collect the temperature data of each measuring point of the machine tool and the real-time thermal error data of the machine tool. The temperature data were measured by a PT100 high-precision patch sensor with a resolution of 0.15 °C and an industrial non-contact infrared probe measuring instrument, and a 12-channel MIK-R5012C Asmik paperless recorder. The thermal error data were measured and collected by the UK’s XL-80 laser interferometer produced by Renishaw Company.

First, temperature measuring points were arranged on each part of the machine tool bed. This paper mainly discusses the influence of the heat generated by the internal heat source of the feed system on the thermal error. The main internal heat sources of a machine tool feed system include the motor, motor coupling, ball screw, rotating nut, bearing seat, guide rail, etc. The heat generated by friction of bearings, screws, rotating nuts, and motors lead to the axial thermal deformation of screws, which directly affects the accuracy of the machine tool feed system [33]. Friction heat generation of the guide rail and working table causes the temperature of the guide rail, bed, and working table to rise, then leads to the elevation and skew of the workbench, and then has a certain impact on the machining accuracy. In addition, if the ambient temperature of the workshop changes too much, it also causes an uneven temperature rise in all parts of the machine tool, which lead to a change in the shape accuracy and machining accuracy of the machine tool, so the ambient temperature is also a consideration [34]. So, in this paper, temperature sensors were arranged at 12 points, such as the motor, bearing, nut, ball screw, guide rail, bed, and worktable of the machine tool; including the ambient temperature, there are a total of 13 temperature variables. Since too many temperature measurement points would affect the model effect of the thermal error prediction modeling process in the next stage, the FCMCA was used to filter the temperature measurement points, select the thermal key sensitive points, and then use the temperature data of the thermal critical sensitive points as the input of the thermal error prediction model for model training.

**Figure 6:**
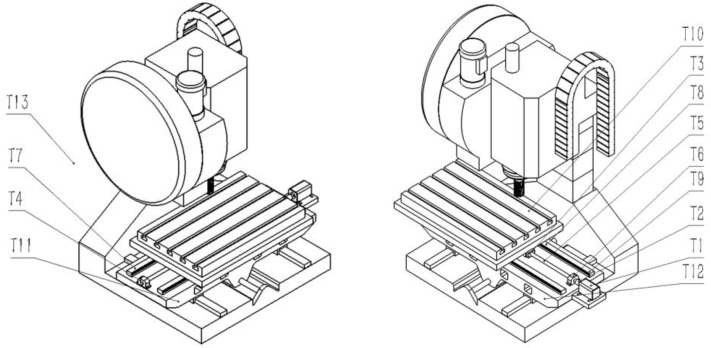
Layout of machine tool temperature measuring points.

**Figure 7:**
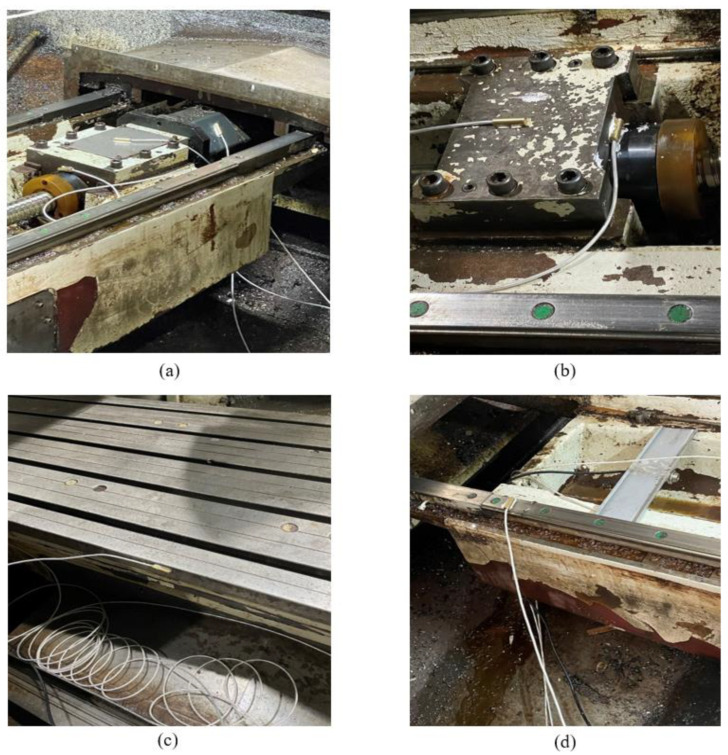
Some site diagrams of temperature measuring points layout and data collection: (**a**) Motor, Motor coupling, Right screw seat, (**b**) Left screw seat, (**c**) Workbench, and (**d**) Left guide rail.

**Figure 8:**
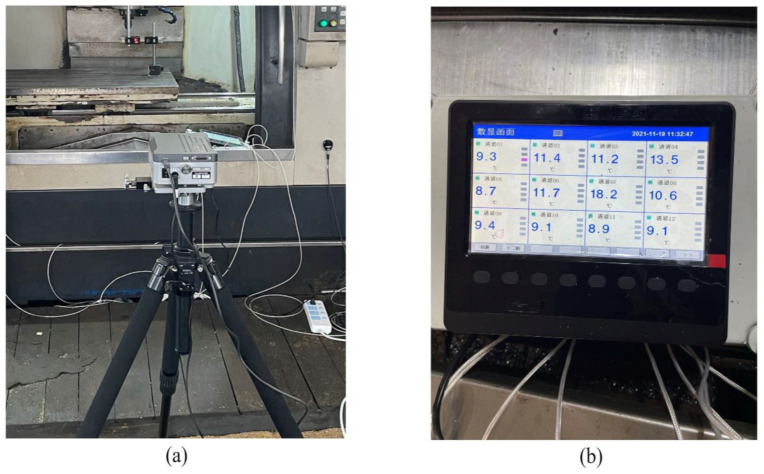
Data acquisition instruments: (**a**) the laser interferometer, (**b**) the paperless recorder.

The layout of temperature measuring points is shown in Figure 6. The specific location description is shown in Table 1. Some of the working scenarios for the arrangement of temperature measurement points and data acquisition are shown in Figure 7. The laser interferometer that collects thermal error data and the 12-channel paperless recorder that collects temperature data are shown in Figure 8. The machine tool used in this article is not very new equipment, but, every year, we carry out equipment overhaul and maintenance of CNC machine tools, as well as system upgrades and other operations to maintain the running state of the machine tools and ensure the machining accuracy of the machine tools. For the friction and wear heat generated by old machine tools, heat dissipation, air cooling, and liquid cooling are usually adopted at the internal heat source of the machine tool to absorb the heat emitted by the heat source so that the temperature can be controlled within a reasonable range, which will not cause excessive thermal deformation of the machine tool, thus avoiding inaccurate thermal deformation analysis and ensuring the universality of the experiment.

**Figure 9:**
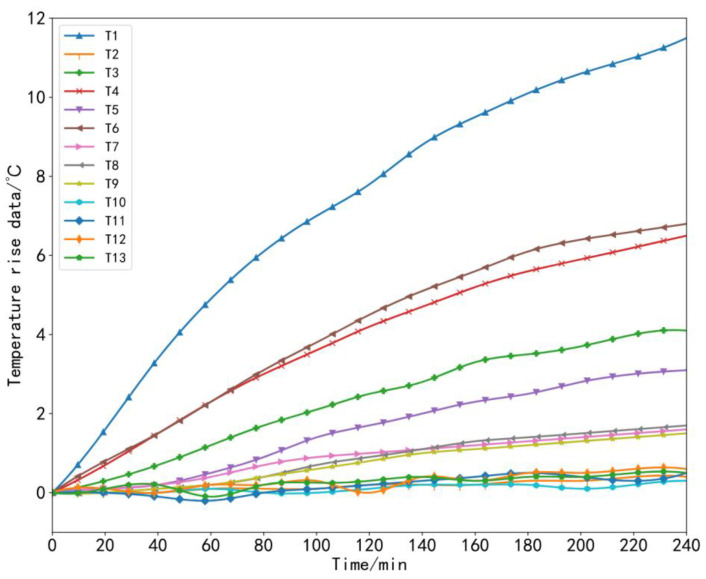
Temperature rise data at a speed of 2000 mm/min.

**Figure 10:**
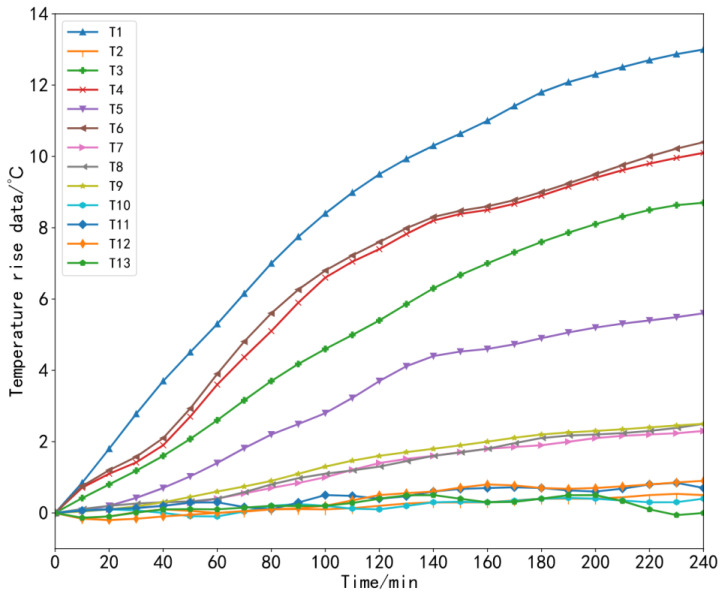
Temperature rise data at a speed of 5000 mm/min.

**Figure 11:**
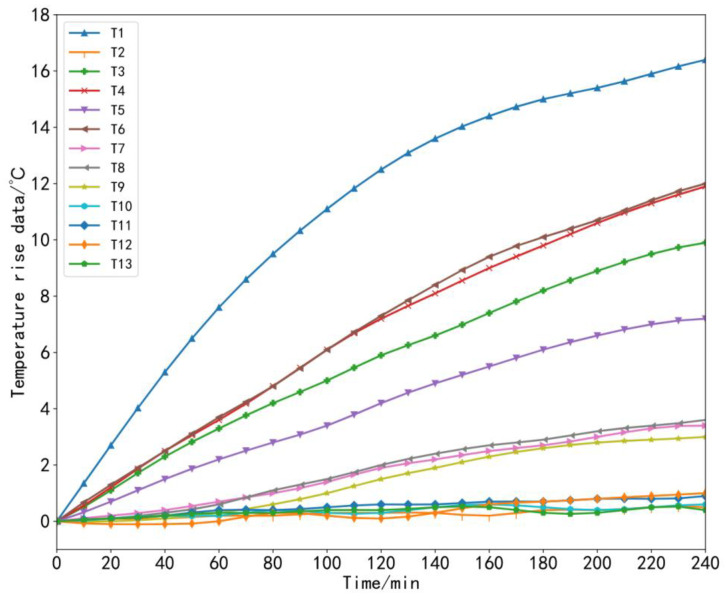
Temperature rise data at a speed of 8000 mm/min.

**Figure 12:**
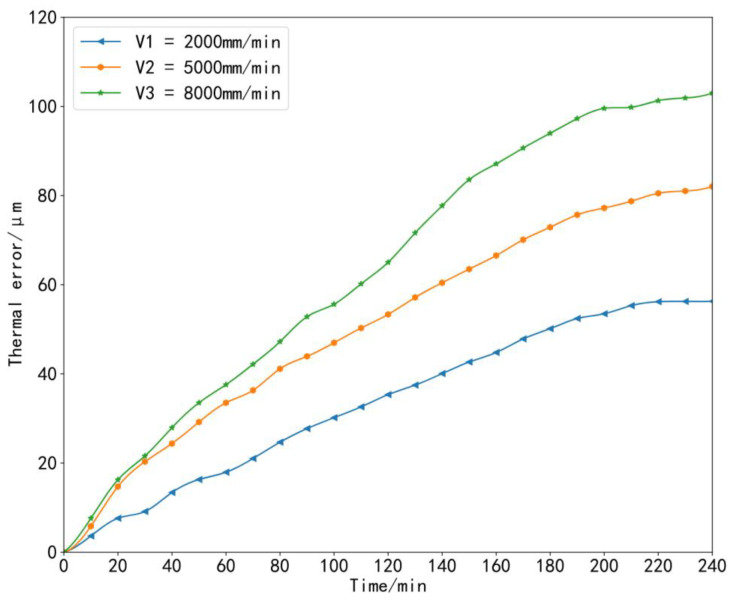
Thermal error data curves at three speeds.

To ensure the generalization and accuracy of the model, it was necessary to collect machine tool temperature data and thermal error data under different working conditions [35]. During the experiment, the machine tool data at three speeds were collected, that is, three sets of thermal error experiments were carried out in total. In the first set of experiments, starting from the cold state of the machine tool at time 0, we set the X-axis of the machine tool to perform linear motion at a feed speed of 2000 mm/min and let the machine tool run continuously for 4 h without load, which was recorded as *V*_1_ = 2000 mm/min. In the second set of experiments, the machine tool still started from a cold state, and the feed rate of the X-axis of the machine tool was set to 5000 mm/min for linear motion; the machine tool ran continuously without load for 4 h, which was recorded as *V*_2_ = 5000 mm/min. In the third set of experiments, other conditions remained unchanged: the machine tool still started from a cold state, the X-axis feed rate was set to 8000 mm/min, and the machine tool was also allowed to run continuously for 4 h without load, which was recorded as *V*_3_ = 8000 mm/min. The temperature and thermal error data were recorded during each experiment every 1 min. In this way, 240 sets of data samples were obtained for each set of experiments. The collected temperature rise data and thermal error data under three working conditions are shown in Figure 9, Figure 10, Figure 11 and Figure 12.

## 4. Establishment of the Thermal Error Prediction Model

**Figure 13:**
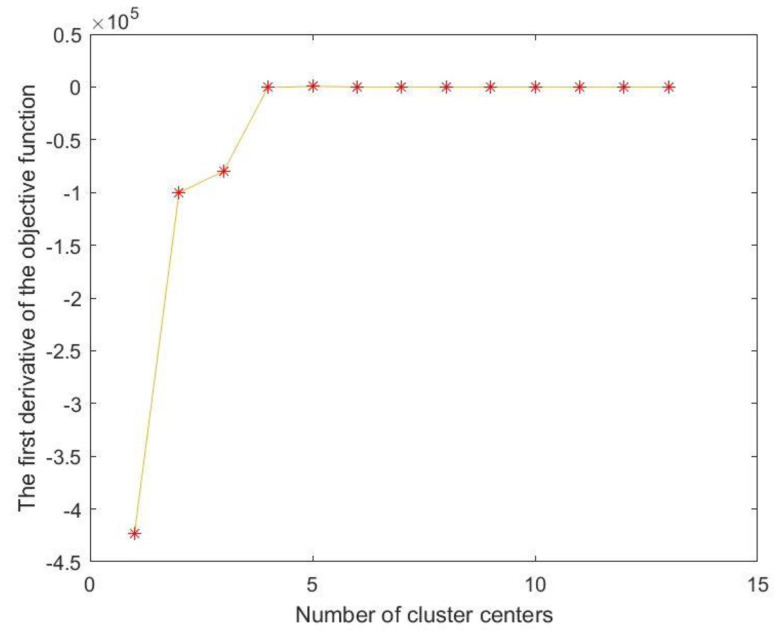
First derivative graph of the objective function.

First, the above FCMCA was used to filter the temperature-sensitive points in the training set. Since a total of 13 temperature measurement points were collected in this experiment, 13 fuzzy clustering analyses needed to be performed. The number of cluster centers was adjusted from 1 to 13 each time, the objective function value was calculated for each clustering, and the first derivative of the objective function was calculated separately. The trend diagram of the first derivative is shown in Figure 13. It can be seen from Figure 13 that when the number of cluster centers is 4, the value of the first derivative of the objective function first approaches 0, so the number of cluster centers is *C* = 4. The fuzzy membership matrix generated when the cluster center was 4 is shown in Table 2.

**Figure 14:**
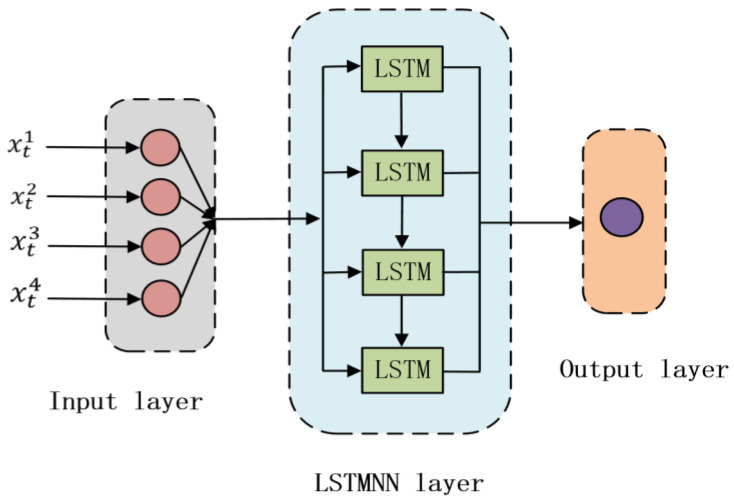
Model network structure diagram.

According to Table 2, T1 to T13 represent 13 temperature measuring points from 1 to 13 from left to right. It can be seen from Table 2 that the generation matrix meets the constraints of the FCMCA, and the sum of the values of each column is approximately equal to 1. The measuring point corresponding to the column with the largest membership coefficient *C(i,j)* in each row of the table is the selected thermal critical sensitive measuring point. The columns corresponding to the maximum values in each row are T1, T4, T8, and T12. Therefore, the four key temperature-sensitive points finally selected were T1, T4, T8, and T12. Next, the temperature rises data of the selected four thermal key sensitive points were used as the input of the SSA-LSTMNN thermal error prediction model, and the thermal error data were used as the output of the model for thermal error prediction modeling. The network structure of SSA-LSTMNN thermal error prediction model is shown in Figure 14, which adopts a three-layer network structure, including an input layer, a hidden layer, and an output layer.

During the experiment, the experimental data of *V*_1_ = 2000 mm/min were used as the training set, and the experimental data of *V*_2_ = 5000 mm/min and *V*_3_ = 8000 mm/min were used as the test set to analyze and establish the thermal error prediction model. Before the input data are sent to the model, normalization is performed to quantify the input data to the interval [0, 1]. The magnitude of input data is unified so that the preprocessed data are limited to a certain range, thus eliminating the adverse effects caused by singular sample data. After the data are normalized, they can speed up the gradient descent to find the optimal solution and improve the model efficiency. Here, Min-Max normalization was used, and the normalization formula is as follows:

(22) $$u_{norm}=\frac{u-u_{min}}{u_{max}-u_{min}}$$

where $u_{norm}$ is the normalized value of each sample, u is the original data value of each sample, $u_{max}$ is the maximum value in the original data sample, and $u_{min}$ is the minimum value in the original data sample.

## 5. Performance Analysis of Thermal Error Prediction Model

In order to further illustrate the superiority of the SSA-LSTMNN thermal error prediction model proposed in this paper, its prediction performance was compared with three other neural network thermal error prediction models. The three models used for comparative analysis were the PSOA-LSTMNN, LSTMNN, and TRNN models. The data sets of all models are consistent with the SSA-LSTMNN model. The prediction performance of each model was verified using test set data at different speeds, *V*_2_ and *V*_3_.

**Figure 15:**
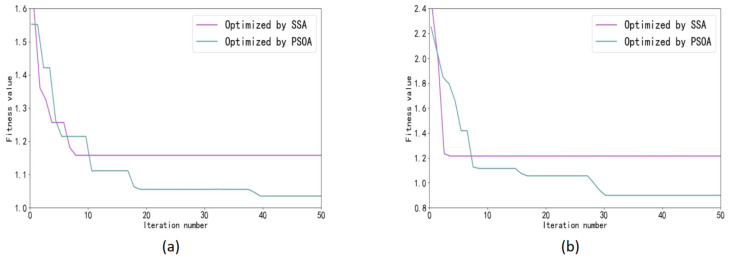
Optimal fitness value curve: (**a**) *V*_2_ = 5000 mm/min best fitness value curve, (**b**) *V*_3_ = 8000 mm/min best fitness value curve.

Both the SSA and PSOA are parameter optimization algorithms used to optimize neural networks, and as explained above, their fitness functions and the number of iterations of the algorithms are the same. After training, the convergence curves of the fitness functions of the two are shown in Figure 15. Figure 15 shows that the number of iterations required to reach the steady state is much smaller than that of the PSOA for the SSA at different speeds, and the values of the SSA after stabilization are higher than those of the PSOA, indicating that the optimization performance of the SSA is higher than that of the PSOA.

Next, we evaluated the robustness of different models using four typical evaluation functions, namely, *Root Mean Squared Error* (*RMSE*), *Mean Absolute Error* (*MAE*), *R-Squared* $(R^{2}$) and *Mean Squared Error* (*MSE*).

*RMSE* represents the sample standard deviation of the difference (called residual) between the predicted value and the measured value. *RMSE* indicates the degree of dispersion of the sample. When doing the nonlinear fitting, the smaller the *RMSE*, the better. *MAE* refers to the average value of the absolute error between the predicted value and the measured value, which can better reflect the actual situation of the error of the predicted value. It is more explanatory and easier to understand. The smaller the *MAE* value is, the smaller the error is. The normal value range of $R^{2}$ is [0, 1], and the closer it is to 1, the stronger the explanatory power of variables to functions, and the better the effect of the model on data fitting. *MSE* refers to the expected value of the square of the difference between the predicted value and the actual value. The smaller the expected value, the closer the predicted value is to the actual value. The calculation formula for each evaluation index is as follows:

(23) $$RMSE=\sqrt{\frac{\sum_{i=1}^{n}{\left(y_{i}-{\hat{y}}_{i}\right)}^{2}}{n}}\;\;\;\;\;i=1,2,\ldots ,n$$

(24) $$MAE=\frac{1}{n}\sum_{i=1}^{n}\left|\left(y_{i}-{\hat{y}}_{i}\right)\right|\;\;\;\;\;i=1,2,\ldots ,n$$

(25) $$R^{2}=1-\frac{\sum_{i=1}^{n}{\left|y_{i}-{\hat{y}}_{i}\right|}^{2}}{\sum_{i=1}^{n}{\left|y_{i}-{\bar{y}}_{i}\right|}^{2}}$$

(26) $$MSE=\frac{1}{n}\sum_{i=1}^{n}{\left(y_{i}-{\hat{y}}_{i}\right)}^{2}$$

Among them, *n* is the total number of thermal errors, *i* is the serial number of thermal errors, $y_{i}$ is the actual value of thermal errors, and ${\hat{y}}_{i}$ is the predicted value of thermal errors. ${\bar{y}}_{i}$ is the average thermal error.

**Figure 16:**
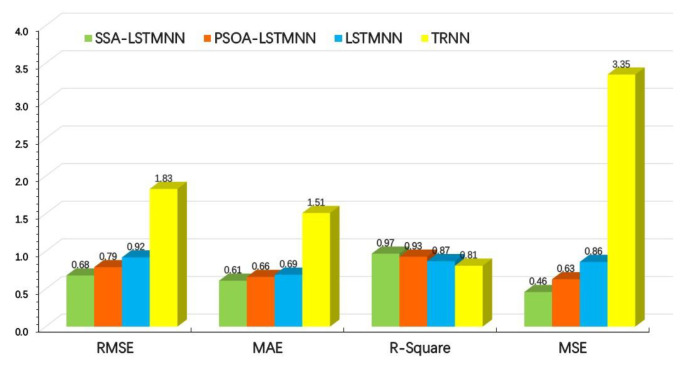
Evaluation results of each model at velocity *V*_2_ = 5000 mm/min.

**Figure 17:**
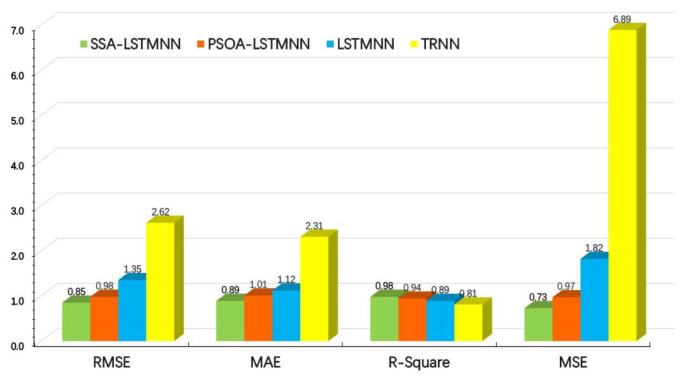
Evaluation results of each model at velocity *V*_3_ = 8000 mm/min.

After the training of models, the results of four evaluation indexes corresponding to the test set data of machine tool at two speeds are shown in Figure 16 and Figure 17.

From the above two graphs, we can see that, at the speed of *V*_2_ = 5000 mm/min, the *RMSE* values of SSA-LSTMNN, PSOA-LSTMNN, LSTMNN, and TRNN are 0.68, 0.79, 0.92 and 1.83, respectively; the *MAE* values are 0.61,0.66, 0.69 and 1.51, respectively; the *R-Squared* values were 0.97, 0.93, 0.87 and 0.81, respectively; and the *MSE* values are 0.46, 0.63, 0.86, and 3.35, separately. That is to say, compared with the other three models, the *RMSE* value of the SSA-LSTMNN model was 14%, 26%, and 63% lower than that of the PSOA-LSTMNN, LSTMNN, and TRNN, respectively; the *MAE* value decreased by 8%, 12%, and 60%, respectively; the *R-Squared* value increased by 4.3%, 11.5%, and 19.7%, separately; and the *MSE* value decreased by 27%, 47%, and 86%, respectively. At the speed of *V*_3_ = 8000 mm/min, the *RMSE* values of the SSA-LSTMNN, PSOA-LSTMNN, LSTMNN, and TRNN are 0.85, 0.98, 1.35, and 2.62, respectively; the *MAE* values are 0.89, 1.01, 1.12, and 2.31, separately; the *R-Squared* values are 0.98, 0.94, 0.89, and 0.81, respectively; and the *MSE* values are 0.73, 0.97, 1.82, and 6.89, separately. Compared with the PSOA-LSTMNN, LSTMNN, and TRNN models, the *RMSE* value of the SSA-LSTMNN model decreased by 13%, 37%, and 67%, respectively; the *MAE* value decreased by 12%, 21%, and 61%, respectively; the value of *R-Squared* increased by 4.2%, 10.1%, and 21%, separately; and the *MSE* value decreased by 25%, 60%, and 89%, respectively. The average *RMSE* values of the SSA-LSTMNN, PSOA-LSTMNN, LSTMNN, and TRNN thermal error prediction models at two different speeds are 0.76, 0.88, 1.13, and 2.22, separately. The average *MAE* values are 0.75, 0.83, 0.9, and 1.91, respectively. The average *R-Squared* values are 0.97, 0.93, 0.88, and 0.81, respectively. The average *MSE* values are 0.59, 0.8, 1.34, and 5.12, separately. Compared with the three other models, the *RMSE* mean value of the SSA-LSTMNN model decreased by 14%, 33%, and 66%; the mean *MAE* value decreased by 9.6%, 16.7%, and 61%; the mean value of *R-Squared* increased by 4.3%, 11.4%, and 10.2%, respectively; and the mean *MSE* value decreased by 26%, 56%, and 88%, respectively.

The above data analysis shows that the performance and fitting effect of the SSA-LSTMNN and PSOA-LSTMNN models are better than those of the LSTMNN and TRNN models, while the SSA-LSTMNN model is even better trained and more accurate than the PSOA-LSTMNN model. This shows that the SSA-LSTMNN thermal error prediction model has an absolute advantage in the field of predicting thermal errors with the highest accuracy and reliability.

**Figure 18:**
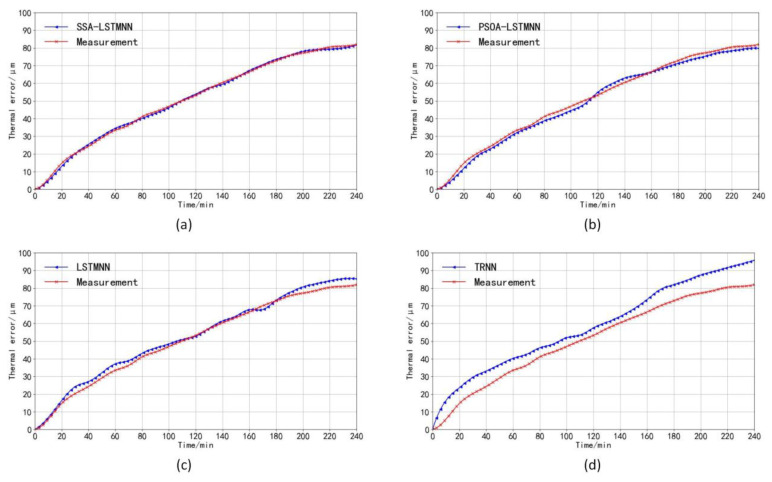
The prediction curve of each model at the speed of 5000 mm/min compared with the measured curve: (**a**) SSA-LSTMNN model, (**b**) PSOA-LSTMNN model, (**c**) LSTMNN model, and (**d**) TRNN model.

**Figure 19:**
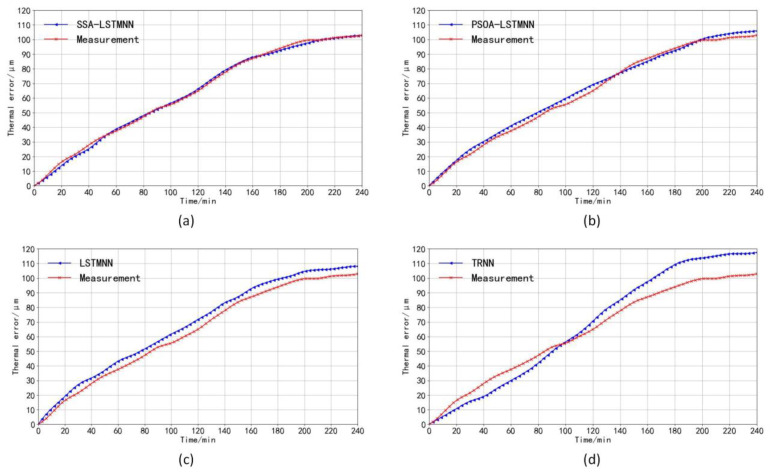
The prediction curve of each model at the speed of 8000 mm/min compared with the measured curve: (**a**) SSA-LSTMNN model, (**b**) PSOA-LSTMNN model, (**c**) LSTMNN model, and (**d**) TRNN model.

**Figure 20:**
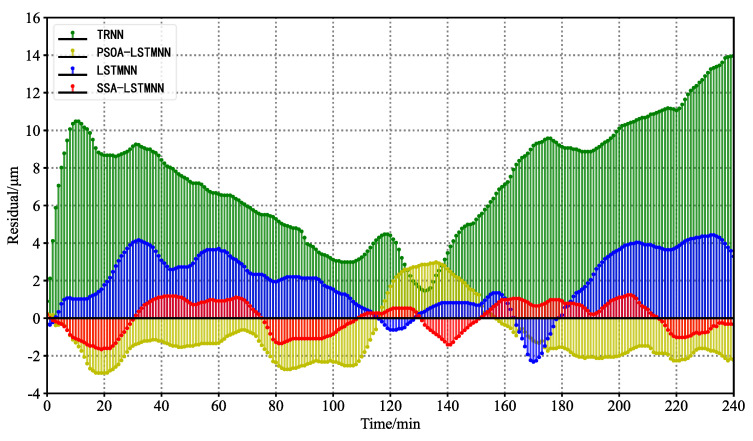
Residual curves for each model at velocity *V*_2_ = 5000 mm/min.

**Figure 21:**
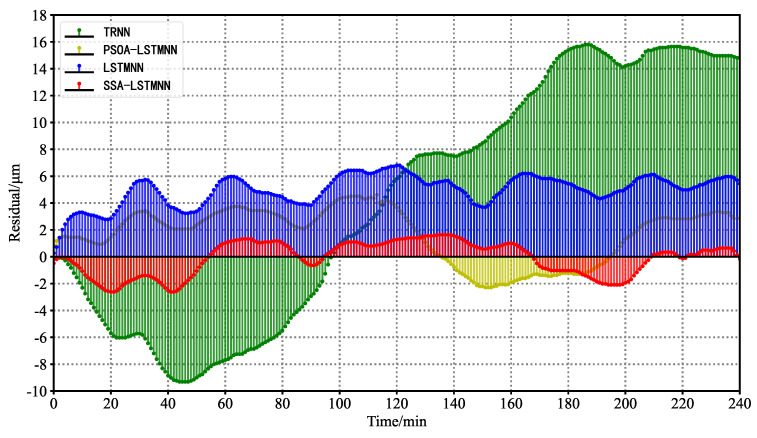
Residual curves for each model at velocity *V*_3_ = 8000 mm/min.

**Figure 22:**
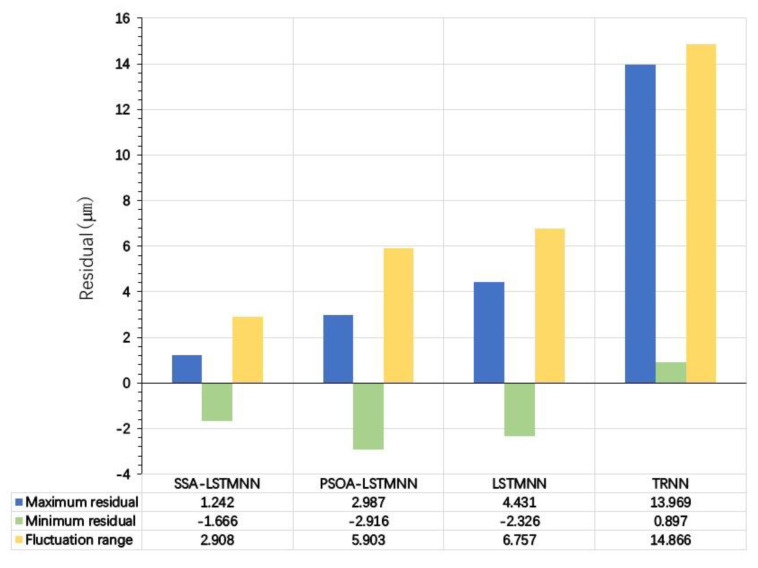
Residual details of each model at speed *V*_2_ = 5000 mm/min.

**Figure 23:**
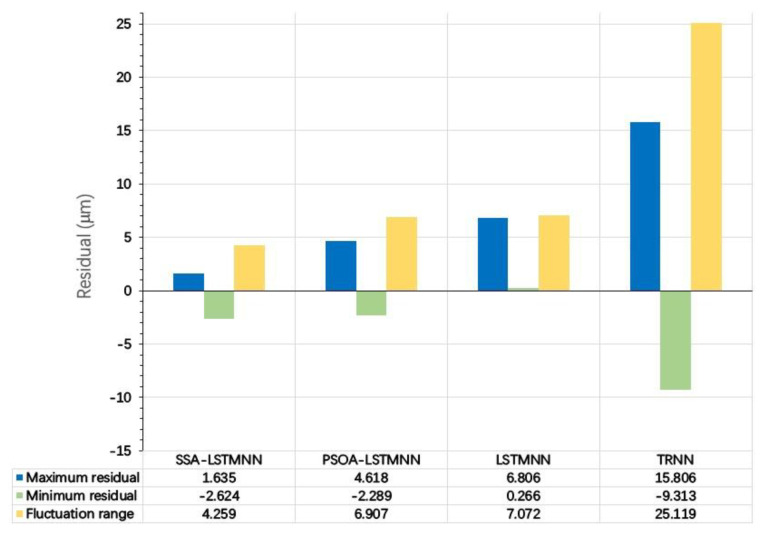
Residual details of each model at speed *V*_3_ = 8000 mm/min.

The prediction curves of thermal error for each prediction model at medium speed 5000 mm/min and high speed 8000 mm/min are shown in Figure 18 and Figure 19. As can be seen in the two figures, the thermal error curves plotted by the SSA-LSTMNN predicted values are the closest to the actual measured thermal error curves at each speed. The predicted curves of the TRNN model deviate the most from the actual error value curves. The fitting degree between the predicted and actual value curves of the PSOA-LSTMNN and LSTMNN models is between that of the SSA-LSTMNN and TRNN. The residual curves and residual details of the four models at different speeds are shown in Figure 20, Figure 21, Figure 22 and Figure 23.

Figure 20 and Figure 21 are residual curves of four thermal error prediction models under two working conditions As can be seen from the figure, the TRNN model has the largest residual coverage and deviates the farthest from the central horizontal axis with the vertical coordinate 0. The LSTMNN model deviates the second farthest from the central axis. The SSA-LSTMNN model residuals are closest to the central axis overall.

As can be seen from Figure 22, the highest value of the predicted residuals of the SSA-LSTMNN thermal error prediction model is 1.242 μm and the lowest value is −1.666 μm at the state of speed *V*_2_ = 5000 mm/min; that is, the maximum deviation of the predicted value curve from the actual value curve is 1.242 μm in the positive direction and 1.666 μm in the negative direction. The fluctuation range of the residuals does not exceed 2.908 μm. The prediction results of the PSOA-LSTMNN model show that its maximum residual value is 2.987 μm and the minimum value is −2.916 μm, and the fluctuation range of the residuals is less than 5.903 μm; the maximum residual value of the predicted value of the LSTMNN model is 4.431 μm and the minimum residual is −2.326 μm, and the fluctuation is not more than 6.757 μm; and the maximum and minimum residual values of the predicted result of the TRNN model predictions are 13.969 μm and 0.897 μm, with fluctuations of no more than 14.866 μm.

Figure 23 depicts that under the condition that the velocity is V_3_ = 8000 mm/min, the maximum residual value of the SSA-LSTMNN model is 1.635 μm, the minimum residual value is −2.624 μm, and the residual variation range is less than 4.259 μm. The highest and lowest residuals of the PSOA-LSTMNN, LSTMNN, and TRNN models are 4.618 μm, −2.289 μm, 6.806 μm, 0.266 μm, 15.806 μm, and −9.313 μm, respectively, with the residual variation ranges not exceeding 6.907 μm, 7.072 μm, and 25.119 μm, separately.

In summary, it can be seen that the SSA-LSTMNN model has the smallest residual and the smallest range of residual variation, indicating that the predicted value of this model is the closest to the measured value among all models, which proves the effectiveness and superiority of the SSA-LSTMNN thermal error prediction model proposed in this paper.

## 6. Conclusions

How to improve the performance of CNCME and the thermal error of machine tools has been a long-term problem in the industrial field. In this paper, a thermal error prediction method is proposed under the premise of addressing this difficult problem. The specific work contents are as follows:

1. Firstly, the FCMCA was used to reduce the 13 temperature measuring points of the CNCME to 4, which eliminates the collinearity problem caused by excessive redundant data of the CNCME temperature, reduces the calculation amount of the thermal error prediction modeling process, and improves the model accuracy.
2. Secondly, the SSA-LSTMNN model was used to train the temperature rise data of key temperature-sensitive points and the thermal error data collected in real-time to build the thermal error prediction model. The long short-term memory recurrent neural network differs from the traditional recurrent neural network in that it takes into account the influence of the temperature rise data of the current moment and the historical moment on the thermal error of the machine tool, which ensures the accuracy of the prediction results. The sparrow search algorithm allows the LSTMNN to be modeled with optimal parameters, optimizing the performance of the LSTMNN and thus enhancing the robustness and strengthening the stability of the model.
3. Finally, the experimental validation was performed under three different working conditions of the machine tool and compared with the PSOA-LSTMNN, LSTMNN, and TRNN prediction models. The final results show that the SSA-LSTMNN model outperforms the other three models in the evaluation of performance metrics such as *RMSE*, *MAE*, *R*^2^, and *MSE*, and the average of the fluctuation range of the residual values of the thermal error prediction results of the SSA-LSTMNN model on two different speed test sets is 44%, 48%, and 82% lower than that of the PSOA-LSTMNN, LSTMNN, and TRNN, respectively. The experimental outcomes demonstrate that the proposed SSA-LSTMNN model in this paper achieves good results; we verified its applicability and its possible generalization, thus showing that the model has a certain industrial application value and is a research prospect in the field of CNCME.

## Figures and Tables

**Table 1:** Description of location of temperature measuring points.

Serial Number of the Temperature Measuring Point	Location of the Temperature Measuring Point
T1	Motor
T2	Motor coupling
T3	Swivel nut
T4, T5, T6	Left screw seat, ball screw, right screw seat
T7, T8, T9	Left guide rail, guide rail, right guide rail
T10	Workbench
T11, T12	Bed left, bed right
T13	Ambient temperature

**Table 2:** Fuzzy membership matrix.

*C(i,j)*	T1	T2	T3	T4	T5	T6	T7	T8	T9	T10	T11	T12	T13
center 1	0.000234	0.001224	0.335729	0.998207	0.035849	0.982883	0.013371	0.007053	0.011702	0.001914	0.982883	0.000951	0.002788
center 2	0.998523	0.000334	0.031218	0.000502	0.005748	0.006271	0.003097	0.001539	0.002659	0.000559	0.006271	0.000274	0.000757
center 3	0.000518	0.981659	0.136941	0.000429	0.075572	0.003759	0.291295	0.087032	0.209473	0.075572	0.978889	0.988877	0.957454
center 4	0.000725	0.016783	0.496113	0.000862	0.882832	0.007088	0.692237	0.904376	0.776166	0.882832	0.018639	0.009898	0.039001

## Data Availability

The data presented in this study are available on request from the corresponding author. They are restricted to experimental results.
