# Correction: Gao et al. A Modeling Method for Thermal Error Prediction of CNC Machine Equipment Based on Sparrow Search Algorithm and Long Short-Term Memory Neural Network. *Sensors* 2023, *23*, 3600

**DOI:** 10.3390/s24072133

**Published:** 2024-03-27

**Authors:** Ying Gao, Xiaojun Xia, Yinrui Guo

**Affiliations:** 1 School of Computer Science and Technology, University of Chinese Academy of Sciences, Beijing 100049, China; xiaxj@sict.ac.cn (X.X.); guoyinrui21@mails.ucas.ac.cn (Y.G.); 2 Shenyang Institute of Computing Technology, Chinese Academy of Sciences, Shenyang 110168, China; 3 School of Mathematics and Computer Sciences, Chifeng University, Chifeng 024000, China

## Error in Figure

In the original publication [1], there was a mistake in Figures 16 and 17. There was a calculation error in the data presented in Figures 16 and 17. The correct Figure 16 and Figure 17 appear below.

**Figure 16:**
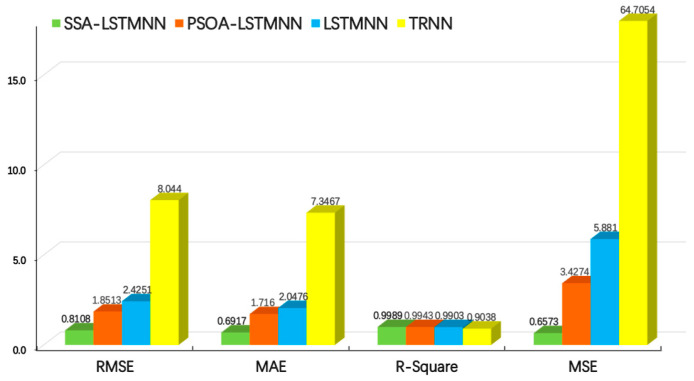
Evaluation results of each model at velocity *V*_2_ = 5000 mm/min.

**Figure 17:**
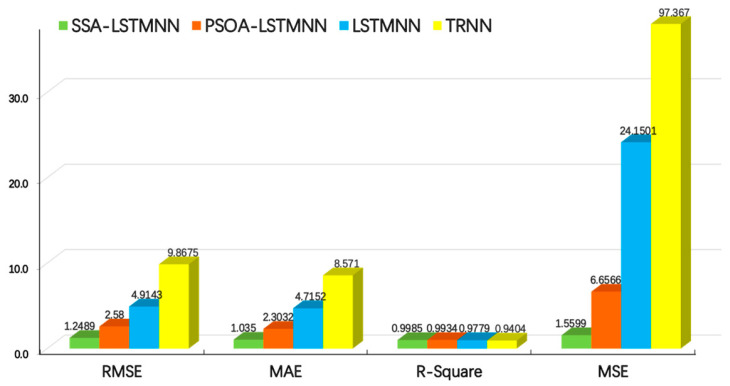
Evaluation results of each model at velocity *V*_3_ = 8000 mm/min.

## Text Correction

There was an error in the original publication [1]. Due to the calculation error, the seventh paragraph of the fifth section of the paper needs to be revised.

A correction has been made to Section 5. Performance Analysis of Thermal Error Prediction Model, Paragraph 7, as follows:

From the above two graphs, we can see that, at the speed of *V*_2_ = 5000 mm/min, the *RMSE* values of SSA-LSTMNN, PSOA-LSTMNN, LSTMNN, and TRNN are 0.8108, 1.8513, 2.4251, and 8.044, respectively; the *MAE* values were 0.6917,1.716, 2.0476, and 7.3467, respectively; the *R-Squared* values were 0.9989, 0.9943, 0.9903, and 0.9038, respectively; and the *MSE* values were 0.6573, 3.4274, 5.881, and 64.7054, respectively. That is to say, compared with the other three models, the *RMSE* value of the SSA-LSTMNN model is 56%, 66%, and 89% lower than that of PSOA-LSTMNN, LSTMNN, and TRNN, respectively; the *MAE* value decreased by 59%, 66%, and 90%, respectively; the *R-Squared* value increased by 0.46%, 0.86%, and 10.52%, respectively; and the *MSE* value decreased by 80%, 88%, and 98%, respectively. At the speed of *V*_3_ = 8000 mm/min, the *RMSE* values of SSA-LSTMNN, PSOA-LSTMNN, LSTMNN, and TRNN are 1.2489, 2.58, 4.9143, and 9.8675, respectively; the *MAE* values were 1.035, 2.3032, 4.7152, and 8.571, respectively; the *R-Squared* values are 0.9985, 0.9934, 0.9779, and 0.9404, respectively; and the *MSE* values were 1.5599, 6.6566, 24.1501, and 97.367, respectively. Compared with the PSOA-LSTMNN, LSTMNN, and TRNN models, the *RMSE* values of the SSA-LSTMNN model decreased by 51%, 74%, and 87%, respectively; the *MAE* value decreased by 55%, 78%, and 88%, respectively; the value of *R-Squared* increased by 0.51%, 2.1%, and 6.17%, respectively; and the *MSE* value decreased by 76%, 93%, and 98%, respectively. The average *RMSE* values of the SSA-LSTMNN, PSOA-LSTMNN, LSTMNN, and TRNN thermal error prediction models at two different speeds are 1.0298, 2.2156, 3.6697, and 8.9557, respectively. The average *MAE* values are 0.8633, 2.0096, 3.3814, and 7.9588, respectively. The average *R-Squared* values are 0.9987, 0.9938, 0.9841, and 0.9221, respectively. The average *MSE* values are 1.1086, 5.042, 15.0155, and 81.0362, respectively. Compared with the three other models, the *RMSE* mean value of the SSA-LSTMNN model decreased by 53%, 71%, and 88%; the mean *MAE* value decreased by 57%, 74%, and 89%; the mean value of *R-Squared* increased by 0.49%, 1.48%, and 8.3%, respectively; and the mean *MSE* value decreased by 78%, 92%, and 98%, respectively.

The authors state that the scientific conclusions are unaffected. This correction was approved by the Academic Editor. The original publication has also been updated.
